# The Acceptable Haemoglobin’s Drop after Uncomplicated Caesarean Sections

**DOI:** 10.34763/jmotherandchild.20242801.d-24-00050

**Published:** 2025-03-21

**Authors:** Basel M. Khreisat, Ainur Donayeva, Zainab Abdul Ameer Jaafar, Zhangeldy Shaimbetov, Ibrahim A. Abdelazim, Zhanslu Sarkulova, Marat Sarkulov, Ainur Omarova

**Affiliations:** Department of Obstetrics and Gynecology, King Hussain Medical Center, Royal Medical Services, Amman, Jordan; Department of Normal Physiology, West Kazakhstan Marat Ospanov Medical University, Aktobe, Kazakhstan; Department of Obstetrics and Gynecology, College of Medicine, Al-Mustansiriya University, Baghdad, Iraq; Pavlodar Region Branch of the Social Health Insurance Fund, Pavlodar, Kazakhstan; Department of Obstetrics and Gynecology, Ain Shams University, Cairo, Egypt; Department of Anesthesiology & Reanimatology, West Kazakhstan Marat Ospanov Medical University, Aktobe, Kazakhstan; Departments of Urology and Andrology, West Kazakhstan Marat Ospanov Medical University, Aktobe, Kazakhstan

**Keywords:** Hemoglobin, Drop, Uncomplicated, Cesarean Sections

## Abstract

**Background:**

Routine haemoglobin assay is a common postoperative (PO) practice after caesarean sections (CSs). There is no consensus regarding the acceptable haemoglobin’s (Hb’s) drop after uncomplicated CSs. Objective: To detect the acceptable Hb’s drop after uncomplicated caesarean sections (CSs).

**Material and Methods:**

Seventy-five (75) participants delivered by uncomplicated elective CSs (ECSs) were recruited for the current study. Participants’ pre-operative Hb was compared to PO-Hbs using t-test to detect the acceptable Hb’s drop after uncomplicated ECSs. Correlations between the 48-hrs. PO-Hb’s drop or 1-week PO-Hb’s drop and estimated blood loss (EBL) during uncomplicated ECSs was detected using Pearson’s correlation.

**Results:**

Mean duration of uncomplicated ECS was 43.3 ± 1.7 min. and mean EBL during uncomplicated ECSs was 654.4 ± 54.49 ml. No significant difference was detected in this study when 48-hrs. PO-Hb (10.89 ± 0.43 gms%) or 1-week PO-Hb (10.86 ± 0.46 gms%) were compared to the participants’ pre-operative Hb (12.2 ± 0.46 gms%), (p=0.3 and 0.5, respectively).

Mean 48-hrs. and 1-week PO-Hb’s drop after uncomplicated ECSs were 1.28 ± 0.09 and 1.3 ± 0.07 gms%, respectively. The correlation between 48-hrs. PO-Hb’s drop and EBL during uncomplicated ECSs was not significant (r=−0.14; p=0.23). The correlation between 1-week PO-Hb’s drop and EBL during uncomplicated ECSs was also not significant (r=0.017; p=0.89).

**Conclusions:**

Mean 48-hrs. and 1-week PO-Hb’s drop after uncomplicated ECSs were 1.28 ± 0.09 and 1.3 ± 0.07 gms%, respectively. Correlations between either the 48-hrs. PO-Hb’s drop or 1-week PO-Hb’s drop and EBL during uncomplicated ECSs were not significant.

## Introduction

Blood loss is one of the major challenges faced during caesarean sections (CSs) [1,2]. Uterine perfusion during pregnancy (750 ml/min) [3] explains the blood loss during CSs [4]. Most of the time, Obstetricians underestimate the blood loss during CSs [5]. Underestimation of the blood loss that occurs during CSs may lead to post-operative (PO) anaemia. Consequently, PO anaemia may lead to defective CS wound healing and postpartum maternal disability [6].

Routine haemoglobin assay is a common PO practice after CSs [7]. Researchers considered blood loss <750 ml, an acceptable blood loss during CS [7,8].

There is no consensus regarding the acceptable Hb’s drop after uncomplicated CSs. Hence, this observational study aimed to detect the acceptable haemoglobin’s (Hb’s) drop after uncomplicated caesarean sections (CSs).

## Material and Methods

Seventy-five (75) participants delivered by uncomplicated elective CSs (ECSs) were recruited for the current observational study. The current study was carried out over the year 2021, **after approval of the Obstetrics and Gynaecology department of Ahmadi Hospital, Kuwait Oil Company (KOC), (Approval No: Ob_0211_20) and written informed consents.**

Inclusion criteria include 1) women admitted for ECSs for their first delivery due to abnormal foetal presentation or abnormal foetal lie (breech presentation or transverse lie), 2) women refused a trial of labour (TOL) after one previous CS and delivered by ECSs, and 3) women admitted for ECSs after ≥ two previous CSs [10,11].

Exclusion criteria: Anaemic participants (Hb <10.9 gms%), placenta previa, twin pregnancies, participants diagnosed with hypertension, diabetes, bleeding, coagulation and/or thrombo-embolism disorders with pregnancy.

Uncomplicated CS means CS with less than 45 min. duration, blood loss less than 750 ml, without any intra-operative complications such as bladder, intestinal, and/or uterine vessel injury [7].

Participants were evaluated using thorough history and abdominal and ultrasound examinations to detect the foetal position, amniotic fluid (AF) volume, placental site and foetal lie before the ECSs.

All ECSs were done under spinal anaesthesia. After the opening of the skin (i.e., Joel-Cohen incision) [12] and abdominal wall layers, the peritoneum over the lower uterine segment (LUS) was opened and dissected downwards [13].

The LUS was incised transversely, followed by drainage of the AF using suction and delivery of the foetus. An intravenous oxytocin (20 IU, Novartis, UK) was given by intravenous infusion after clamping of the umbilical cord (i.e., 3^rd^ stage management) [14]. The LUS incision was repaired after delivery of the placenta using polyglycolic sutures (i.e., Ethicon, USA) in 2 layers.

Estimated blood loss (EBL) during ECSs was calculated by the blood’s quantity in the suction bottle and post-operative towels’ weight - pre-operative towels’ weight [15,16].

Blood samples were collected 48 hrs. after the ECSs from the studied participants for PO-Hb assay according to the hospital’s protocol [17] and one week after ECSs following Carson et al.’s [18] recommendations.

Participants’ pre-operative Hb was compared to PO-Hbs to detect Hb’s drop after uncomplicated ECSs (primary outcome). The correlations between either the 48-hrs. PO-Hb’s drop or 1-week PO-Hb’s drop and EBL during uncomplicated ECSs was detected using Pearson’s correlation (secondary outcome).

## Sample size and Statistical analysis

The G Power 3.1.9.4 (Düsseldorf; Germany) with 0.05 α-error probability and 0.95% power was used to calculate the sample size for this study [19,20,21]. Participants’ pre-operative Hb was compared to PO-Hbs using *t*-test to detect the acceptable Hb’s drop after uncomplicated ECSs. The correlations between either the 48-hrs. PO-Hb’s drop or 1-week PO-Hb’s drop and EBL during uncomplicated ECSs was detected using Pearson’s correlation. p< 0.05 was considered significant.

## Results

Seventy-five (75) participants delivered by uncomplicated ECS were recruited for the current observational study. Participants’ pre-operative Hb was compared to PO-Hbs to detect the Hb’s drop after uncomplicated ECSs. The correlations between either the 48-hrs. PO-Hb’s drop or 1-week PO-Hb’s drop and EBL during uncomplicated ECSs was detected using Pearson’s correlation.

The mean participant age was 27.1 ± 2.8 years, and the mean gestational age at ECSs was 38.42 ± 0.29 gestational weeks. About 18.7% (14/75) of the uncomplicated ECSs were primi-ECSs, while 81.3% (61/75) of them were ERCSs. The mean duration of uncomplicated ECS was 43.3 ± 1.7 min. and mean EBL during uncomplicated ECSs was 654.4 ± 54.49 ml. Table 1.

**Table 1:** Participants’ characteristics, types and duration of ECSs, EBL, pre-operative and PO-Hbs

**Variables**	**Studied participants (Number 75)**	***p-*value (95% CI)**
Maternal age (Years)	27.1 ± 2.8	
Gestational age at ECS (Weeks’)	38.42 ± 0.29	
BMI (Kg/m^2^)	27.92 ± 2.16	
**Type of the ECS**		
-Primi-ECS	14/75 (18.7%)	
-ERCS	61/75 (81.3%)	
ECS duration (min.)	43.3 ± 1.7	
Estimated intra-operative blood loss (ml)	654.4 ± 54.49	
**Pre-operative Hb (gms%)**	**12.2 ± 0.46**	**0.3 (1.17, 1.3, 1.5)**
48-hours PO-Hb (gms%)	10.89 ± 0.43	
48-hours PO-Hb drop	1.28 ± 0.09	
**Pre-operative Hb (gms%)**	**12.2 ± 0.46**	**0.5 (1.2, 1.3, 1.5)**
1-week PO-Hb (gms%)	10.86 ± 0.46	
1-week PO-Hb drop	1.3 ± 0.07	

BMI: Body mass index. CI: Confidence interval. Data presented as number and percentage (%) and mean ± standard deviation (SD). EBL: Estimated blood loss. ECS: Elective cesarean section. ERCS: Elective repeat cesarean section. Hb: Hemoglobin. PO: Post-operative. *t*-test was used for statistical analysis to compare the 48-hrs and 1-week PO-Hbs to pre-operative Hb.

### The acceptable haemoglobin’s drop after uncomplicated ECSs

Although, the 48-hrs. and 1-week PO-Hbs were less than the participants’ pre-operative Hb in this study, this difference was statistically insignificant. Additionally, no significant difference was detected in this study when the 48-hrs. PO-Hb (10.89 ± 0.43 gms%) or 1-week PO-Hb (10.86 ± 0.46 gms%) were compared to the participants’ pre-operative Hb (12.2 ± 0.46 gms%), [p= 0.3 (95%CI; 1.17, 1.3, 1,5) and 0.5 (95%CI; 1.2, 1.3, 1.5); respectively]. Table 1.

The mean 48-hrs. and 1-week PO-Hb’s drop after uncomplicated ECSs were 1.28 ± 0.09 and 1.3 ± 0.07 gms; respectively. Table 1.

### Relation between PO-Hb’s drop and EBL during uncomplicated ECSs

**Figure 1.**
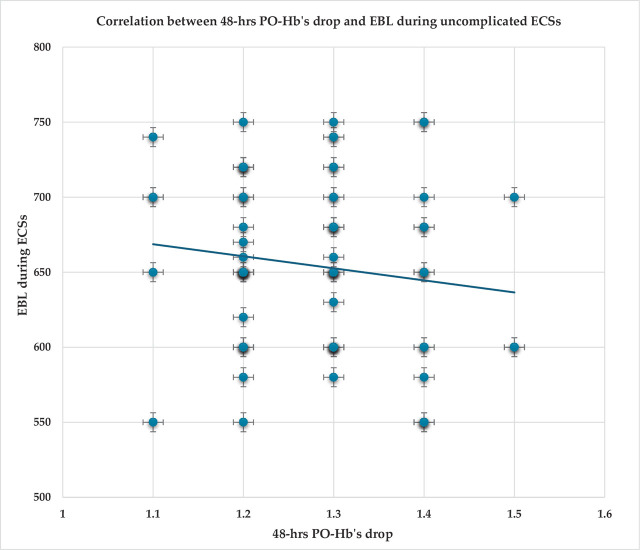
Correlation between 48-hrs. post-operative hemoglobin’s (PO-Hb’s) drop and estimated blood loss (EBL) during uncomplicated elective cesarean sections (ECSs).

The correlation between 48-hrs. PO-Hb’s drop and EBL during uncomplicated ECSs was not significant (r =−0.14; p=0.23). Figure 1.

**Figure 2.**
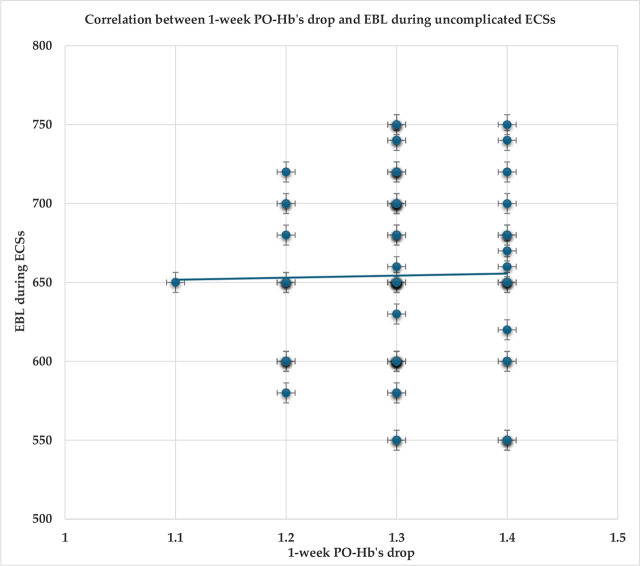
Correlation between 1-week postoperative hemoglobin’s (PO-Hb’s) drop and estimated blood loss (EBL) during uncomplicated elective cesarean sections (ECSs).

The correlation between 1-week PO-Hb’s drop and EBL during the uncomplicated ECSs was also not significant (r =0.017; p=0.89). Figure 2.

## Discussion

Researchers considered blood loss <750 ml an acceptable blood loss during CS [7,8]. There is no consensus regarding the acceptable Hb’s drop after uncomplicated CSs. Hence, this observational study aimed to detect the acceptable haemoglobin’s drop after uncomplicated caesarean sections.

Participants’ pre-operative Hb was compared to PO-Hbs to detect the Hb’s drop after uncomplicated CSs. The correlations between either the 48-hrs. PO-Hb’s drop or 1-week PO-Hb’s drop and EBL during uncomplicated CSs was detected using Pearson’s correlation.

Mean participants’ gestational at ECSs was 38.42 ± 0.29 gestational weeks. Most of the Obstetricians prefer to do the ECS after 38 gestational weeks following the ACOG recommendation [22].

Although, the WHO recommends that the CS rate in any country should not exceed 10–15% of all deliveries [23]. About 18.7% (14/75) of the studied ECSs were primi-ECSs, while 81.3% (61/75) of them were ERCSs. This may be due to increased CS rate worldwide (30% in the United States, 52.2% in Cyprus and 24.6% in England [4,23]) following maternal request and extensive foetal monitoring [22].

Mean duration and mean EBL during uncomplicated ECS in this study were 43.3 ± 1.7 min. and 654.4 ± 54.49 ml, respectively.

Uncomplicated CS means CS with less than 45 min. duration, blood loss less than 750 ml, without any intra-operative complications such as bladder, intestinal, or uterine vessel injury [7].

A randomized controlled trial (RCT) found the mean operative time was the same (51 min.) for either primi-ECSs or ERCSs [17]. The mean operative time and EBL for the uncomplicated CSs were 43.1 ± 7.2 min. and 485.9 ± 136.7 ml; respectively in an observational study [4].

Bodur et al. [7], reported an average EBL of 517.1 ± 417.6 ml for either primi or repeat uncomplicated CSs, and researchers considered blood loss <750 ml, an acceptable blood loss during CS [8].

### The acceptable Hb’s drop after uncomplicated ECSs

A prospective observational study used the 48-hrs. PO-Hb to detect the PO-Hb’s drop after CSs [17]. Carson et al. [18] recommend 1-week PO-Hb to detect the transfusion risks following hip surgeries [18].

Therefore, the participants’ pre-operative Hb was compared to both the 48-hrs. and 1-week PO-Hbs in the current study, which aimed to detect the acceptable haemoglobin’s drop after uncomplicated caesarean sections.

Although the 48-hrs. and 1-week PO-Hbs were less than the participants’ pre-operative Hb in this study, this difference was statistically insignificant. Additionally, no significant difference was detected in this study when the 48-hrs. PO-Hb (10.89 ± 0.43 gms%) or 1-week PO-Hb (10.86 ± 0.46 gms%) were compared to the participants’ pre-operative Hb (12.2 ± 0.46 gms%), (p=0.3 and 0.5; respectively).

An observational study failed to find any statistical difference, when the pre-operative Hb was compared to either 48-hrs. PO-Hb or 1-week PO-Hb after uneventful/uncomplicated CSs [4].

Bodur et al. [7], found the average pre-operative and PO-Hbs after uncomplicated CSs were 12.09 ± 0.18 and 10.72 ± 1.39 gms%, respectively.

A prospective observational study also found the average pre-operative and PO-Hbs after uncomplicated CSs were 12.23 ± 1.13 and 10.74 ± 1.49 gm%, respectively [17].

In this study, the mean 48-hrs. and 1-week PO-Hb’s drop after uncomplicated CSs were 1.28 ± 0.09 and 1.3 ± 0.07 gms, respectively.

Bodur et al. [7] found the average PO-Hb’s drop after uncomplicated CSs was 1.36 ± 1.06 gms% [7] and a prospective observational study found the average post-CS Hb’s drop after uncomplicated CSs was 1.52±1.27 gm% [17].

Another retrospective study found the average PO-Hb’s drop after uncomplicated CSs was 1.3 ± 0.67 gms% [24].

### Relation between PO-Hb’s drop and EBL during uncomplicated ECSs

The correlation between 48-hrs. PO-Hb’s drop and EBL during uncomplicated ECSs was not significant (r=−0.14; p=0.23). The correlation between 1-week PO-Hb’s drop and EBL during uncomplicated ECSs was also not significant (r=0.017; p=0.89).

Similarly, the correlation between the PO-Hb’s drop and EBL during uncomplicated CSs was not significant in an observational study [4].

This was the first observational research that aimed to detect the acceptable Hb’s drop after uncomplicated CSs. No significant difference was detected in this study when the 48-hrs. PO-Hb (10.89 ± 0.43 gms%) or 1-week PO-Hb (10.86 ± 0.46 gms%) were compared to the participants’ pre-operative Hb (12.2 ± 0.46 gms%). The mean 48-hrs. and 1-week POHb’s drop after uncomplicated CSs were 1.28 ± 0.09 and 1.3 ± 0.07 gms, respectively. The correlations between either the 48-hrs. PO-Hb’s drop or 1-week PO-Hb’s drop and EBL during uncomplicated ECSs were not significant. Further randomized trials with good sample sizes are needed to detect the acceptable Hb’s drop after complicated and uncomplicated CSs.

### Key points

- Routine haemoglobin assay is a common PO practice after CSs. There is no consensus regarding the acceptable Hb’s drop after uncomplicated CSs.
- No significant difference was detected in this study when the 48-hrs. PO-Hb (10.89 ± 0.43 gms%) or 1-week PO-Hb (10.86 ± 0.46 gms%) were compared to the participants’ pre-operative Hb (12.2 ± 0.46 gms%).
- The mean 48-hrs. and 1-week PO-Hb’s drop after uncomplicated CSs were 1.28 ± 0.09 and 1.3 ± 0.07 gms, respectively. Correlations between either the 48-hrs. PO-Hb’s drop or 1-week PO-Hb’s drop and EBL during uncomplicated ECSs were not significant.
